# Structural controllability of general edge dynamics in complex network

**DOI:** 10.1038/s41598-023-30554-7

**Published:** 2023-02-28

**Authors:** Shaopeng Pang, Yue Zhou, Xiang Ren, Fangzhou Xu

**Affiliations:** 1 School of Information and Automation Engineering, Qilu University of Technology (Shandong Academy of Sciences), Jinan, 250353 China; 2 Second Academy of China Aerospace Science and Industry Corporation, Beijing, 100143 China; 3 International School for Optoelectronic Engineering, Qilu University of Technology (Shandong Academy of Sciences), Jinan, 250353 China

**Keywords:** Statistical physics, thermodynamics and nonlinear dynamics, Complex networks

## Abstract

Dynamic processes that occur on the edge of complex networks are relevant to a variety of real-world systems, where states are defined on individual edges, and nodes are active components with information processing capabilities. In traditional studies of edge controllability, all adjacent edge states are assumed to be coupled. In this paper, we release this all-to-all coupling restriction and propose a general edge dynamics model. We give a theoretical framework to study the structural controllability of the general edge dynamics and find that the set of driver nodes for edge controllability is unique and determined by the local information of nodes. Applying our framework to a large number of model and real networks, we find that there exist lower and upper bounds of edge controllability, which are determined by the coupling density, where the coupling density is the proportion of adjacent edge states that are coupled. Then we investigate the proportion of effective coupling in edge controllability and find that homogeneous and relatively sparse networks have a higher proportion, and that the proportion is mainly determined by degree distribution. Finally, we analyze the role of edges in edge controllability and find that it is largely encoded by the coupling density and degree distribution, and are influenced by in- and out-degree correlation.

## Introduction

Complex networks containing interacting dynamic units are ubiquitous in social, financial and natural systems^1–3^. In recent years, the controllability of complex networks has received extensive attention and research^4–8^. Most studies of network controllability have focused on the nodal dynamics. However, the edge dynamics^9,10^, which are relevant to various real-world systems, are also important in network science. It is suitable for modeling networks where states are defined on individual edges, and nodes are active components with information processing capabilities. A seminal work addressing edge controllability is presented by Nepusz et al.^9^. They introduced the switchboard dynamics (SBD) model to describe the edge dynamics and study its structural controllability. Much interest has been stimulated toward exploring the controllability properties of edge dynamics. Representative studies of edge dynamics include its controllable subspace^11^, target control^12,13^, controllability optimization^14^, robustness^15^, and applications in multi-agent systems^16–18^.

Most of the existing work on the edge controllability describes the edge dynamics based on the SBD. The condition that the SBD requires all adjacent edge states to be coupled is too strong, which makes the SBD have certain limitations and cannot accurately describe various real-world systems. In this paper, we release this all-to-all coupling restriction and propose a general edge dynamics (GED) model that can describe the edge dynamics with arbitrary coupling relationships between edge states, which generalizes the SBD. For example, in the system with computers and routers, edges represent the physical connections such as fiber optics and cables. A node (i.e., a computer or router) continuously processes information received from some of its upstream neighbors and transmits it to some of its downstream neighbors. The information received and transmitted by a node can be represented by the states on its incoming and outgoing edges. The switching matrix in each node controls the dynamic process, and the elements in the switching matrix determine which upstream and downstream neighbors of a node to receive and transmit information. A social network can also be modeled as the GDE, in which people are nodes, the information transmitted between people are the edge states, and the switching matrix in each node controls the reception and transmission of information. The proposal of GED has caused a series of questions, such as the structural controllability of GED, the controllability characteristics, the role of coupling and the role of edges in the edge controllability, etc.

We study the structural controllability of edge dynamics based on the GED. Firstly, we give a theoretical framework to determine the minimum set of driver nodes and driven edges required to fully control the GED. We find that the set of driver nodes for edge controllability is unique and determined by the local information of nodes, which is fundamentally different from nodal controllability. Secondly, we find that the coupling density among edge states plays an important role in edge controllability, where the coupling density is the proportion of adjacent edge states that are coupled. Specifically, there exist lower and upper bounds of edge controllability, which are determined by the coupling density. Meanwhile, there is a vast range between the controllability bounds, in which a broad spectrum of edge controllability can be achieved by adjusting the coupling density. We analyze empirical networks and propose theoretical formulations to demonstrate that the controllability bounds generally exist in the edge controllability of arbitrary networks. Thirdly, we investigate the proportion of effective coupling when controlling the GED. Simulation finds that homogeneous and relatively sparse networks have a higher proportion of effective coupling, and the proportion is mainly determined by the degree distribution. Finally, we analyze the role of edges in edge controllability by classifying each edge into one of three categories: critical, ordinary, and intermittent. We find that the proportions of the three kinds of edges are largely encoded by the coupling density and degree distribution, and are influenced by in- and out-degree correlation.

## General edge dynamics

The SBD^9^ provides a characterization of edge dynamics on a directed network *G*(*V*, *E*). Let $\textbf{y}_v^-$ and $\textbf{y}_v^+$ represent the state vectors corresponding to the incoming and outgoing edge states of node *v*, respectively. The factor that can affect $\textbf{y}_v^+$ is the vector $\textbf{y}_v^-$, the damping vector ${\varvec{\tau }}_v$, and the external input vector $\textbf{u}_v$. Then the switchboard dynamics can be described as:

1 $$\begin{aligned} \dot{\textbf{y}}_v^+=S_v \textbf{y}_v^--{\varvec{\tau }}_v\otimes \textbf{y}_v^+ + \sigma _v\textbf{u}_v, \end{aligned}$$

where $S_v \in \mathbb {R}^{k_v^+\times k_v^-}$ is the ‘switching matrix’. The number of its row and column are equal to the out-degree $k_{v}^{ + }$ and the in-degree $k_{v}^{ - }$ of node *v*, respectively. $\sigma _{v}$ is one if node *v* is a driver node and is zero otherwise. $\otimes$ denotes the entry-wise product of the two vectors of the same size.

A linear time-invariant system can be established by reformulating Eq. (1) in terms of the edge state $x_{i}$, which is

2 $$\begin{aligned} {\dot{\textbf{x}}}=(W-T) \textbf{x}+H {\textbf{u}}, \end{aligned}$$

where $W \in \mathbb {R}^{M \times M}$ is the transpose of the adjacency matrix of the line graph *L*(*G*) of *G*, in which $w_{{ij}}$ is nonzero if and only if the head of edge *j* is the tail of edge *i*. For the line graph *L*(*G*) converted from an original graph *G*, the nodes in *L*(*G*) correspond to the edges in *G*, and an edge in *L*(*G*) corresponds to a length-two directed path in *G*. $T\in \mathbb {R}^{M \times M}$ is a diagonal matrix whose diagonal elements correspond to the damping terms for each edge. Note that *T* can be ignored in the edge controllability^9,19^. $H\in \mathbb {R}^{M \times M}$ is a diagonal matrix where the *i*th diagonal element is $\sigma _{v}$ if node *v* is the tail of edge *i*.

In the study of structural controllability^4,9^, both the state and input matrices are structural matrices, where their elements are either fixed 0 or independent free parameters. A system is called structurally controllable if it is possible to fix the free parameters in the state and input matrices to certain values so that the obtained system is controllable in the usual sense, i.e., the generic rank of the controllability matrix

3 $$\begin{aligned} C=(H,WH,W^2H,...,W^{M-1}H), \end{aligned}$$

has full rank ${\mathrm{rank}}_{\mathrm{g}}(C)=M$, where the generic rank of a structural matrix is the maximal rank that the structural matrix achieves as a function of its free parameters.

**Figure 1:**
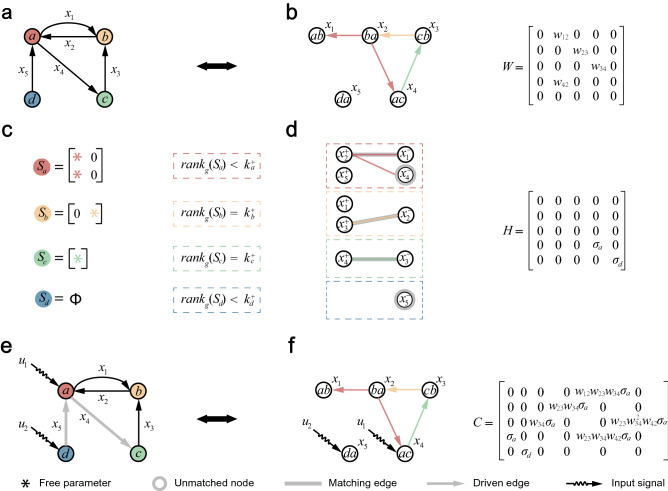
Structural controllability of general edge dynamics. (**a**) A directed network *G* with 4 nodes and 5 edge states $\{x_1, x_2, x_3, x_4, x_5\}$ in GED. (**b**) The trimmed line graph $L(G^{\prime } )$ of *G*. The colors of edges in $L(G^{\prime } )$ correspond to those of the nodes in (**a**). (**c**) The switching matrices $\{S_a, S_b, S_c, S_d\}$ in *G*. (**d**) The bipartite graph *H*(*G*) of $L(G^{\prime } )$ with 4 matching blocks. (**e**) Driver nodes ($v_{a}$ and $v_{d}$), driven edges ($x_{4}$ and $x_{5}$) and input signals ($u_{1}$ and $u_{2}$) for controlling the GED of *G*. (**f**) Driver nodes ($x_4$ and $x_5$) and input signals ($u_1$ and $u_2$) for controlling the nodal dynamics of $L(G')$.

However, the SBD requires all the elements in each switching matrix $S_{v}$ are independent free parameters. The elements in $S_{v}$ capture the coupling relationship between the incoming and outgoing edge states of nodes. The absence of 0 in $S_{v}$ means that the incoming and outgoing edge states of each node are completely coupled. This restrictive conditions are too strong. We release this all-to-all coupling restriction and consider the GED, in which the elements in the switching matrix $S_{v}$ are either fixed 0 or independent free parameters. The fundamental difference between SBD and GED is that GED allows 0 elements in the switching matrix. For example, An GED with 4 nodes and 5 edge states $\left\{ {x_{1} ,x_{2} ,x_{3} ,x_{4} ,x_{5} } \right\}$ is shown in Fig. 1a. Its switching matrices $\left\{ {S_{a} ,S_{b} ,S_{c} ,S_{d} } \right\}$ contain either fixed 0 or independent free parameters.

## Structural controllability of general edge dynamics

We are interested in configuring an appropriate input matrix *H* so that the GED is structurally controllable. The theory^9^ no longer applies to the GED. We thus give a theoretical framework for the structural controllability of the GED, which is quantified based on the minimum number of driver nodes (i.e., the minimum number of nodes with $\sigma _v=1$) and the minimum number of driven edges (i.e., the minimum number of non-zero columns of *H*) required to fully control the GED.

Equation (2) indicates that the GED of a digraph *G* is equivalent to the nodal dynamics of its trimmed line graph $L(G')$, where the nodes in $L(G')$ correspond to the edges in *G*, and an edge in $L(G')$ corresponds to an independent free parameter in the switching matrix of *G*. This equivalence shows that the GED of *G* and the nodal dynamics of $L(G')$ have the same state set and state matrix *W*. For example, as shown in Fig. 1a and c, the GED of a digraph *G* contains 5 edge states $\{x_1, x_2, x_3, x_4, x_5\}$ and 4 switching matrices $\{S_a, S_b, S_c, S_d\}$. Its trimmed line graph $L(G')$ is shown in Fig. 1b, where each independent free parameter in the switching matrix corresponds to a directed edge of $L(G')$. Taking $S_a$ as an example, the independent free parameters in $S_a$ correspond to edges $e_{x_2, x_1}$ and $e_{x_2, x_4}$ in $L(G')$, respectively.

We give the first method for determining the minimum number of driver nodes and driven edges required to fully control the GED. Firstly, applying the minimum input theorem^4^ to the nodal dynamics of $L(G')$ gives us the bipartite graph *H*(*G*). The maximum matching method^4^ can determine the minimum unmatched nodes in *H*(*G*), which correspond to the minimum driver nodes required to control the nodal dynamics of $L(G')$. For example, the nodal dynamics of $L(G')$ and its bipartite graph *H*(*G*) are shown in Fig. 1b and d, respectively. The unmatched nodes $x_4$ and $x_5$ in *H*(*G*) is the driver nodes. How to controlling the nodal dynamics of $L(G')$ is shown in Fig. 1f. Secondly, the driver nodes in the nodal dynamics of $L(G')$ correspond one-to-one with the driven edges in the GED of *G*^9^. This allows us to determine the minimum number of driven edges needed to control the GED of *G*. For example, as shown in Fig. 1e and f, the driver nodes $x_4$ and $x_5$ in the nodal dynamics of $L(G')$ correspond to the driven edges $x_4$ and $x_5$ in the GED of *G*. Thirdly, the driver node required to control the GED of *G* is the starting node of the driven edge. As shown in Fig. 1e, the driver nodes are the starting nodes $v_a$ and $v_d$ of the driven edges. The generic rank of the controllability matrix has full rank ${\mathrm{rank}}_{\mathrm{g}}(C)=5$, indicating that the GED of *G* is structurally controllable. In summary, we can determine the minimum number of driver nodes and driven edges required to control the GED with the help of minimum input theorem.

We further propose a framework to determine the minimum driver nodes and driven edges based on the local information of nodes, since the minimum input theorem is computationally complex and requires global information of network. Firstly, a bipartite graph *H*(*G*) can be partitioned into *N* ‘matching blocks’ according to *N* switching matrices. The matching block corresponding to a switching matrix $S_v$ contains the incoming edge states and outgoing edges edge states of the node *v*, where the incoming edge states and outgoing edges edge correspond to the left and right nodes of the matching block, respectively. Meanwhile the independent free parameters in $S_v$ correspond one-to-one to the edges in the matching block. For example, as shown in Fig. 1c and d, taking $S_a$ as an example, its matching block contains the edge states $\{x_2^+, x_5^+\}$ and $\{x_1^-, x_4^-\}$. The independent free parameters in $S_a$ have a one-to-one correspondence with the edges in its matching block. Note that the incoming (outgoing) edge state of a node in GED is only coupled with the outgoing (incoming) edge state of this node, which means that the edges in the partitioned bipartite graph *H*(*G*) only exist in each matching block, not between matching blocks. As shown in Fig. 1d, no edge spans any two matching blocks. This ensures that the maximum matching result of the partitioned bipartite graph *H*(*G*) remains unchanged. Secondly, we prove that the matching block of $S_v$ contains unmatched nodes if and only if $S_v$ has no full row rank, i.e., ${\mathrm{rank}}_{\mathrm{g}}(S_v)<k_v^+$. Meanwhile, the number of unmatched nodes in the matching block of $S_v$ is equal to $k_v^+-{\mathrm{rank}}_{\mathrm{g}}(S_v)$. The detailed proof process is in Supplementary Note 1. Thirdly, the driver nodes (unmatched nodes) in the nodal dynamics of $L(G')$ correspond one-to-one with the driven edges in the GED of *G*^9^. It can be deduced that, for the structural controllability of the GED of *G*, a node *v* is the driver node if ${\mathrm{rank}}_{\mathrm{g}}(S_v)<k_v^+$, and the number of driven edges in the outgoing edge set of the driver node is $k_v^+-{\mathrm{rank}}_{\mathrm{g}}(S_v)$. As shown in Fig. 1c and d, taking $S_a$ as an example, it is the driver node due to ${\mathrm{rank}}_{\mathrm{g}}(S_a)<k_a^+$. Meanwhile, the number of driven edges in the outgoing edge set of *a* is $k_a^+-{\mathrm{rank}}_{\mathrm{g}}(S_a)=1$.

Based on the above analysis, we present our major conclusions. The minimum number $N_{\mathrm{D}}$ of driver nodes required to control the GED is

4 $$\begin{aligned} N_{\mathrm{D}}=N_{({\mathrm{rank}}_{\mathrm{g}}(S_v)<k_v^+)}+\sum _{i=1}^{N_{\beta }}\beta _i, \end{aligned}$$

where $N_{(x)}$ is the number of nodes that satisfy the condition *x*. $N_{\beta }$ is the number of connected components. $\beta _i=1$ if the connected component is the full-rank component, and $\beta _i=0$ otherwise, where the full-rank component is a strongly connected component with each node in it satisfies $k_v^-=k_v^+={\mathrm{rank}}_{\mathrm{g}}(S_v)$. Note that the accumulation term $\sum _{i=1}^{N_{\beta }}\beta _i$ is to ensure reachability. Specifically, we randomly select a node in each full-rank component as the driver node, and randomly select an outgoing edge of the driver node as the driven edge. Then the minimum number $M_{\mathrm{D}}$ of driven edges is

5 $$\begin{aligned} M_{\mathrm{D}}=\sum _{i=1}^N(k_i^+-{\mathrm{rank}}_{\mathrm{g}}(S_i))+\sum _{i=1}^{N_{\beta }}\beta _i. \end{aligned}$$

The above formulas allow us to determine the minimum set of driver nodes and driven edges required to control the GED based on the local information of nodes. The detailed proof process is in Supplementary Note 1.

## Controllability characteristics

We study the controllability characteristics of GED based on real and model networks. The controllability is quantitatively described by the proportion $n_{\mathrm{D}}=N_{\mathrm{D}}/N$ of driver nodes and the proportion $m_{\mathrm{D}}=M_{\mathrm{D}}/M$ of driven edges required for control. Since the switching matrix of GED contains 0 and independent free parameters, we introduce the coupling density $P \in [0,1]$ to quantify the probability that an element in the switching matrix is an independent free parameter.

**Figure 2:**
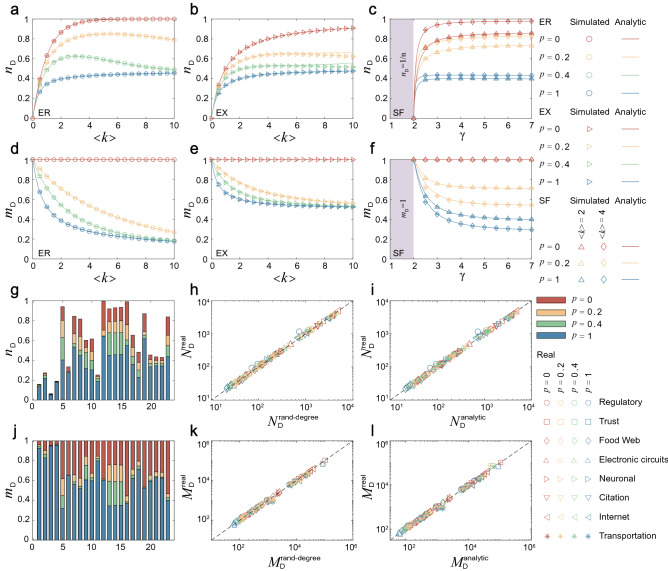
Controllability characteristics. The variation of $n_{\mathrm{D}}$ and $m_{\mathrm{D}}$ in (**a**, **d**) ER networks, (**b**, **e**) EX networks, and (**c**, **f**) SF networks as the function of the coupling density *P*, the average degree $\langle k \rangle$ and the power exponent $\gamma$, respectively. The results of (**g**) $n_{\mathrm{D}}$ and (**j**) $m_{\mathrm{D}}$ in real networks with $P=0$, $P=0.2$, $P=0.4$, and $P=1$, respectively. Numbers refer to the network indices in Table 1. The results of (**h**) $N_{\mathrm{D}}^\mathrm{rand-degree}$ and (**k**) $M_{\mathrm{D}}^\mathrm{rand-degree}$ obtained from the degree-preserving randomized version of the real networks, compared with the real results, respectively. The theoretical predictions of (**i**) $N_{{\mathrm{D}}}^{\mathrm{analytic}}$ and (**l**) $M_{\mathrm{D}}^{\mathrm{analytic}}$ compared with the real results, respectively. All the numerical results are obtained by averaging over 100 independent network realizations.

Figure 2a–f give the variation of $n_{\mathrm{D}}$ and $m_{\mathrm{D}}$ in Erdős-Rényi (ER), exponential (EX) and scale-free (SF) networks according to the coupling density *P*, the average degree $\langle k \rangle$ and the power exponent $\gamma$, respectively. An important finding is that there are upper and lower bounds on $n_{\mathrm{D}}$ and $m_{\mathrm{D}}$. Specifically, when $P=0$, there are no independent free parameters in the switching matrix, that is, there is no coupling between the incoming and outgoing edge states of any node, resulting in $n_{\mathrm{D}}$ and $m_{\mathrm{D}}$ reaching the upper bounds. Conversely, when $P=1$, the incoming and outgoing edge states of each node are coupled, resulting in $n_{\mathrm{D}}$ and $m_{\mathrm{D}}$ reaching the lower bounds. Further, the gaps between the upper and lower bounds are very large, except for the case of small $\langle k \rangle$ and $\gamma$. Any values of $n_{\mathrm{D}}$ and $m_{\mathrm{D}}$ between bounds can be achieved by adjusting *P*. This demonstrates that the coupling density has a significant impact on the edge controllability. Another finding is that $n_{\mathrm{D}}$ of some ER networks shows a non-monotonic change with the increase of $\langle k \rangle$ in Fig. 2a, and larger value of *P* move the peak to the direction where $\langle k \rangle$ increases. The non-monotonic is caused by the different change rate of edges and couplings in these model networks with the change of $\langle k \rangle$. We provide a theoretical analysis of the non-monotonic. The details of the theoretical analysis are shown in model networks.

Figure 2g and j give $n_{\mathrm{D}}$ and $m_{\mathrm{D}}$ of the GED constructed from real network topologies. We find that the results for $n_{\mathrm{D}}$ and $m_{\mathrm{D}}$ vary greatly with the coupling density *P*, but do not exceed the upper bound (when $P=0$) and lower bound (when $P=1$). Another important factor affecting $n_{\mathrm{D}}$ and $m_{\mathrm{D}}$ is the degree distribution. Therefore, we apply a degree-preserving randomization (rand-degree)^4^, which keeps the in- and out-degrees of each node unchanged but reconnects the nodes randomly. As shown in Fig. 2h and k, this procedure does not alter $N_{\mathrm{D}}$ and $M_{\mathrm{D}}$ significantly. Equations (4) and  (5) show that $N_{\mathrm{D}}$ and $M_{\mathrm{D}}$ are determined by the local network information (i.e., in- and out-degrees of each node and coupling density) and the full-rank component. The degree-preserving randomization can keep the local network information unchanged, but it can hardly generate new full-rank components. Therefore, $N_{\mathrm{D}}$ and $M_{\mathrm{D}}$ of a degree-preserving randomization are very close to those of its original network. This indicates that $n_{\mathrm{D}}$ and $m_{\mathrm{D}}$ are determined mainly by the coupling density and degree distribution. Furthermore, we bring the coupling density and the degree distribution of real networks into our theoretical formulas to give analytical predictions of $n_{\mathrm{D}}$ and $m_{\mathrm{D}}$. As shown in Fig. 2i and l, for most real networks, a good agreement is obtained between the analytical predictions and the real results. The values of $n_{\mathrm{D}}$ and $m_{\mathrm{D}}$ of the real network are shown in Table 1. See Supplementary Note 3 for the details of theoretical predictions for real networks.

In summary, we find that the coupling density has a significant impact on the edge controllability, leading to the controllability bounds being pervasive in both model and real networks. We can estimate the edge controllability of the network based on the coupling density and degree distribution.

## The role of coupling

**Figure 3:**
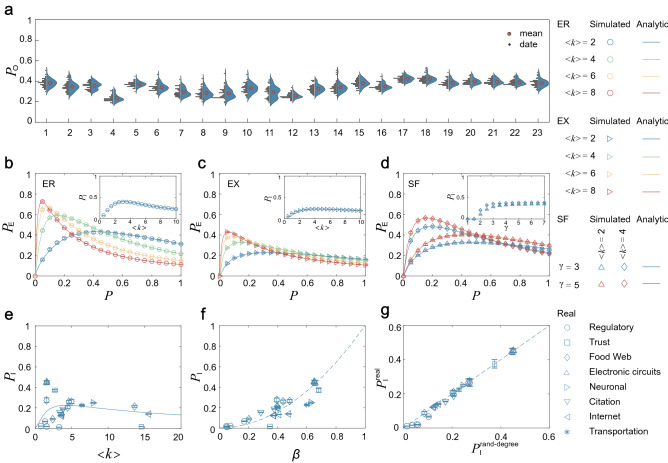
The role of coupling. (**a**) The half violin plot of $P_{{\text{O}}}$ for real networks. The proportion $P_\mathrm{E}$ of effective coupling and its trapezoidal numerical integration $P_{\mathrm{I}}$ in (**b**) ER networks, (**c**) EX networks, and (**d**) SF networks as the function of the coupling density *P*, the average degree $\langle k \rangle$ and the power exponent $\gamma$, respectively. (**e**) The integration $P_{\mathrm{I}}$ of real networks as the function of $\langle k \rangle$. (**f**) The integration $P_{\mathrm{I}}$ of real networks as the function of the correlation parameter $\beta$. (**g**) The integration $P_{\mathrm{I}}^\mathrm{rand-degree}$ obtained from the degree-preserving randomized version of the real networks, compared with $P_{\mathrm{I}}^\mathrm{real}$.

We study the role of coupling (i.e., independent free parameter) on the edge controllability. Firstly, we gradually increase the coupling density *P* until the number $M_{\mathrm{D}}$ of driven edges required to control the GED reaches the lower bound. Let the coupling density at this time be $P_{{\text{O}}}$, as shown in Fig. 3a, we find that the $P_{{\text{O}}}$ of most real networks is not high. This shows that most of the coupling are ineffective, that is, their absence does not affect the edge controllability.

This inspires us to study the proportion of effective coupling. Specifically, for a node *v*, we delete the independent free parameters in its switching matrix $S_v$ one by one under the premise that the number of driven edges in its outgoing edge set remains unchanged. We find that the minimum number of independent free parameters is equal to the generic rank of the switching matrix. Therefore, the proportion of effective coupling is

6 $$\begin{aligned} P_{\mathrm{E}}=\frac{1}{N}\sum _{i=1}^{N}{\mathrm{rank}}_{\mathrm{g}}(S_i)/(k_i^+ k_i^-P), \end{aligned}$$

where $k_i^+ k_i^-P$ is the number of independent free parameters in the switching matrix of a node, and ${\mathrm{rank}}_{\mathrm{g}}(S_i)$ is the minimum number of independent free parameters that keeps the number of driven edges in the outgoing edge set of this node unchanged. Figure 3b–d give the variation of $P_{{\text{E}}}$ in ER, EX and SF networks according to the coupling density *P*, the average degree $\langle k \rangle$ and the power exponent $\gamma$, respectively. An important finding is that $P_{{\text{E}}}$ and *P* are not positively correlated, as $P_{{\text{E}}}$ decreases with increasing *P* when *P* is large enough. This confirms the conclusion that $P_{{\text{O}}}$ is not high in real networks. Another interesting phenomenon is that $P_{{\text{E}}}$ shows a distinct peak for a specific value of *P*. Both the location and the height of the peak mainly depend on $\langle k \rangle$, and larger value of $\langle k \rangle$ moves the peak to the direction where *P* decreases.

To further study the effect of network topology on $P_{{\text{E}}}$, we give the trapezoidal numerical integral of $P_{{\text{E}}}$ in the interval [0, 1], denoted as $P_{\mathrm{I}}$. The integral operation allows $P_{\mathrm{I}}$ to be independent of *P* and is only relevant to the network topology. As shown in the panels in Fig. 3b–d, we find that homogeneous (i.e., big $\gamma$) and relatively sparse (i.e., small $\langle k \rangle$) networks have the highest $P_{\mathrm{I}}$. Then we analyze the effect of network topology on $P_{\mathrm{I}}$ based on real networks. On the one hand, we give the dependence of $P_{\mathrm{I}}$ of real networks on $\langle k \rangle$. As shown in Fig. 3e, $P_{\mathrm{I}}$ of relatively sparser real networks is higher, which confirms the results based on model networks. On the other hand, we give the dependence of $P_{\mathrm{I}}$ of real networks on the correlation parameter^6^, i.e., $\ \beta =1-\frac{1}{2M}\sum _{i}|k_i^+ -k_i^-|$. The parameter $\beta \in [0, 1]$ captures the in- and out-degree correlation of a network. For example, $\beta = 1$ indicates the perfect positive correlation between in- and out-degrees of nodes, i.e., $k_v^+=k_v^-$ for each node. As shown in Fig. 3f, there is a basic positive correlation between $P_{\mathrm{I}}$ and $\beta$, which further states that the homogeneous networks have higher $P_{\mathrm{I}}$. To analyze the dependence of $P_{\mathrm{I}}$ in the real network, we apply degree-preserving randomization. As shown in Fig. 3g, this procedure does not alter $P_{\mathrm{I}}$ significantly. This indicates that $P_{\mathrm{I}}$ is determined mainly by the degree distribution. In other words, $P_{\mathrm{I}}$ is determined mainly by the number of incoming and outgoing edges of each node and is independent of where those edges point. The values of $P_{{\text{O}}}$ and $P_{{\mathrm{I}}}$ of the real network are shown in Table 1.

In summary, we find that most of coupling are ineffective in edge controllability. Homogeneous and relatively sparse networks have a higher proportion of effective coupling. The proportion of effective coupling is mainly determined by the degree distribution.

## The role of edges

We explore the role of edges in edge controllability by classifying each edge into one of three categories^20^: critical, ordinary, and intermittent. Specifically, critical means that an edge must be the driven edge; ordinary means that it is not the driven edge and intermittent means that it belongs to the set of driven edges with a certain probability. We neglect the possible presence of full-rank component, which is uncommon in directed networks and has little effect on the number $M_{\mathrm{D}}$ of driven edges. Then the category of an edge depends solely on the switching matrix of its source node. Specifically, the number of driven edges in the set of outgoing edges of a driver node *v* is $k_v^+-{\mathrm{rank}}_{\mathrm{g}}(S_v)$. Therefore, the judgment methods of the three kinds of edges are as follows:

1. An edge is critical if it corresponds to an all-zero row in the switching matrix of its source node. In particular, an edge is critical if its source node satisfies $k_v^+ > k_v^- = 0$.
2. An edge is ordinary if ${\mathrm{rank}}_{\mathrm{g}}(S_v)>{\mathrm{rank}}_{\mathrm{g}}(S'_v)$, where $S_v$ is the switching matrix of the source node *v* of the edge, and $S'_v$ is the switching matrix of *v* after deleting the non-zero row corresponding to the edge.
3. An edge is intermittent if ${\mathrm{rank}}_{\mathrm{g}}(S_v)={\mathrm{rank}}_{\mathrm{g}}(S'_v)$.

**Figure 4:**
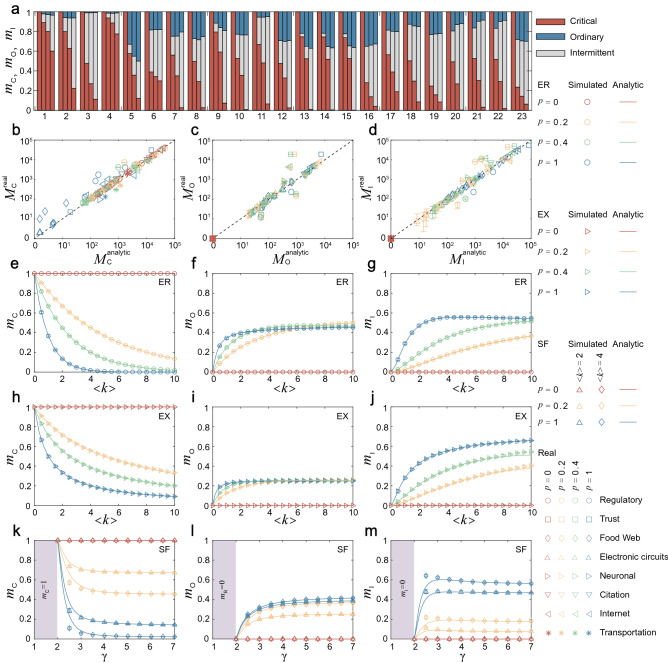
The Role of Edges. (**a**) The proportion of critical $m_\mathrm{C}$, ordinary $m_\mathrm{O}$, and intermittent $m_{\mathrm{I}}$ edges in each real network with the coupling density $P=0$, $P=0.2$, $P=0.4$, and $P=1$, respectively. The proportion (**b**) $M_\mathrm{C}^\mathrm{analytic}$, (**c**) $M_\mathrm{O}^\mathrm{analytic}$, and (**d**) $M_{\mathrm{I}}^\mathrm{analytic}$ obtained by theoretical prediction, compared with the real results. The variation of $m_\mathrm{C}$, $m_\mathrm{O}$, and $m_{\mathrm{I}}$ in (**e**–**g**) ER networks, (**h**–**j**) EX networks, and (**k**–**m**) SF networks as the function of the coupling density *P*, the average degree $\langle k \rangle$ and the power exponent $\gamma$, respectively.

We employ the real and model networks to substantiate the edge categories and offer analytical results. The proportions of critical, ordinary and intermittent edges in each real network with the coupling density $P=0$, $P=0.2$, $P=0.4$, and $P=1$ are shown in Fig. 4a. A notable finding is that as *P* increases, the proportion of critical edges in each real network is drastically reduced and replaced by ordinary and intermittent edges. The reason is that as *P* increases, the all-zero rows in the switching matrix gradually sparse. This facilitates that most real networks are dominated by ordinary and intermittent edges when *P* is large enough. Note that the existence of a few star-shaped giants in the regulatory networks (No. 1 and 4 in Table 1) is responsible for their large number of critical edges.

The way of identifying edges can be used to derive analytical formulas for the expected fraction of three edge categories in real and model networks (see Supplementary Note 2 and 3 for the detailed procedures). As shown in Fig. 4b–d, a good agreement is obtained between the theoretical predictions and the real results. This shows that the proportions of three kinds of edges can be predicted by considering the coupling density and degree distribution. Note that the reason why the theoretical predictions deviate slightly from the real results is that the theoretical predictions are performed by assuming that in- and out-degrees of nodes have no correlation in real networks. Then we give the simulation and analytical results of model networks. One of the main findings is that *P* has a significant impact on the proportion of the three kinds of edges, especially in dense and homogeneous networks. For ER networks in Fig. 4e–g, we find that at low $\langle k \rangle$, the networks are dominated by critical edges. The reason is that abundant nodes without an incoming edge exist in the networks. As the network grows, the fraction of ordinary and intermittent edges increases rapidly. As shown in Fig. 4h–j, the trends of the curves in EX networks are very similar to those provided by ER networks. But the fraction of ordinary edges is smaller, counterbalanced by a greater proportion of critical and intermittent edges. Figure 4k–m give the variation of three kinds of edges in SF networks with $\gamma$. We find that heterogeneous (small $\gamma$) networks are dominated by critical edges. However, when *P* is large enough, the homogeneous network contains very few critical edges, replaced by ordinary and intermittent edges. The values of $m_{{\text{C}}}$, $m_{{\text{O}}}$ and $m_{\mathrm{I}}$ of the real network are shown in Table 1.

**Table 1:** Structural controllability of general edge dynamics in real network. For each network, we obtain its type, number, name, number *N* of nodes, number *M* of edges, the lower bounds $\left( {n_{{\text{D}}}^{{\text{L}}} \;{\text{and}}\;m_{{\text{D}}}^{{\text{L}}} } \right)$, the upper bound $\left( {n_{{\text{D}}}^{{\text{U}}} \;{\text{and}}\;m_{{\text{D}}}^{{\text{U}}} } \right)$, $P_{{\text{O}}}$, $P_{{\text{I}}}$ and the proportions of the three kinds of edge $\left( {m_{{\text{C}}}^{{P = 1}} ,m_{{\text{O}}}^{{P = 1}} ,m_{{\text{I}}}^{{P = 1}} } \right)$.

${\mathrm{Type}}$	${\mathrm{No.}}$	${\mathrm{Name}}$	${\mathrm{N}}$	${\mathrm{M}}$	$n_{\mathrm{D}}^{\mathrm{L}}$	$n_{\mathrm{D}}^{\mathrm{U}}$	$m_{\mathrm{D}}^{\mathrm{L}}$	$P_{\mathrm{O}}$	$P_{\mathrm{I}}$	$m_{\mathrm{C}}^{P=1}$	$m_{\mathrm{O}}^{P=1}$	$m_{\mathrm{I}}^{P=1}$
${\mathrm{Regulatory}}$	1	${\mathrm{Ownership}}-{\mathrm{USCorp}}$^21^	8497	6726	0.136	0.159	0.924	0.384	0.022	0.601	0.034	0.365
	2	${\mathrm{TRN-EC-2}}$ ^22^	423	578	0.220	0.274	0.829	0.348	0.068	0.223	0.062	0.715
	3	${\mathrm{TRN-Yeast-1}}$^23^	4684	15451	0.052	0.064	0.947	0.356	0.011	0.113	0.009	0.878
	4	${\mathrm{TRN-Yeast-2}}$^22^	688	1079	0.177	0.190	0.952	0.236	0.019	0.775	0.011	0.214
${\mathrm{Trust }}$	5	${\mathrm{prison}}$ ${\mathrm{inmate}}$^24,25^	67	182	0.403	0.940	0.319	0.372	0.374	0.121	0.500	0.379
	6	${\mathrm{WikiVote}}$^26^	7115	103689	0.281	0.335	0.653	0.342	0.017	0.299	0.182	0.519
$${\mathrm{Food}}$$$${\mathrm{web}}$$	7	$${\mathrm{St.Marks}}$$^27^	45	224	0.533	0.844	0.563	0.296	0.263	0.027	0.205	0.768
	8	${\mathrm{Seagrass}}$^28^	49	226	0.449	0.816	0.518	0.286	0.262	0.027	0.252	0.721
	9	${\mathrm{grassland}}$^29^	88	137	0.318	0.602	0.606	0.282	0.278	0.073	0.190	0.737
	10	${\mathrm{Ythan}}$^29^	135	601	0.304	0.615	0.597	0.332	0.205	0.008	0.235	0.757
	11	${\mathrm{Silwood}}$^30^	154	370	0.188	0.253	0.797	0.293	0.087	0.065	0.049	0.886
	12	${\mathrm{Little}}$ ${\mathrm{Rock}}$^31^	183	2494	0.639	0.995	0.603	0.258	0.210	0.025	0.292	0.683
${\mathrm{Electronic}}$ ${\mathrm{circuits}}$	13	${\mathrm{S208a}}$^22^	122	189	0.451	0.918	0.344	0.329	0.446	0.011	0.370	0.619
	14	${\mathrm{S420a}}$^22^	252	399	0.456	0.929	0.348	0.341	0.446	0.005	0.366	0.629
	15	${\mathrm{S838a}}$^22^	512	819	0.459	0.934	0.350	0.382	0.452	0.002	0.364	0.634
$\mathrm {Neorunal}$	16	${\mathrm{C.elegans}}$^32^	297	2359	0.549	0.990	0.374	0.349	0.249	0.041	0.326	0.633
${\mathrm{Citation}}$	17	${\mathrm{Scimet}}$^33^	2729	1041	0.360	0.653	0.623	0.424	0.188	0.189	0.200	0.611
	18	${\mathrm{Kohonen}}$^34^	3772	12731	0.230	0.482	0.715	0.424	0.155	0.086	0.149	0.765
${\mathrm{Internet}}$	19	${\mathrm{Political}}$ ${\mathrm{blogs}}$ ^35^	1224	19090	0.619	0.870	0.523	0.388	0.140	0.085	0.222	0.693
	20	${\mathrm{p2p-1}}$^36^	10876	39994	0.334	0.454	0.591	0.398	0.134	0.004	0.121	0.875
	21	${\mathrm{p2p-2}}$^36^	8846	31839	0.344	0.435	0.628	0.397	0.124	0.032	0.085	0.883
	22	${\mathrm{p2p-3}}$^36^	8717	31525	0.343	0.429	0.625	0.391	0.130	0.018	0.087	0.895
${\mathrm{Transportation}}$	23	${\mathrm{USair97}}$^37^	332	2126	0.437	0.837	0.400	0.382	0.252	0.063	0.299	0.638

Note that, when $P=0$, we have $m_{\mathrm{D}}^\mathrm{U}$ = 1, $m_\mathrm{C}^{P=0}=1$, $m_\mathrm{O}^{P=0}=0$, $m_{\mathrm{I}}^{P=0}=0$.

In conclusion, we find that the proportions of three kinds of edges are to a great extent encoded by the coupling density and the degree distribution, and are affected by the in- and out-degree correlation. When the coupling density is large enough, dense and homogeneous networks have lower proportions of critical edges, replaced by ordinary and intermittent edges.

## Conclusion

Most existing research on edge controllability is based on the SBD. However, the SBD requires all adjacent edge states to be coupled. This restrictive conditions are too strong. In this paper, we release this all-to-all coupling restriction and propose the GED, which can describe the edge dynamics with arbitrary coupling relationships between edge states. We give a theoretical framework to study the structural controllability of GED. An important finding is that the set of driver nodes for edge controllability is unique and determined by the local information of nodes, where the local information of a node includes in-degree, out-degree and the generic rank of its switching matrix. Then we find that the coupling density among edge states plays an important role, leading to the lower and upper bounds of edge controllability. At the same time, we can estimate the edge controllability of an arbitrary network based on its coupling density and degree distribution. Furthermore, we investigate the role of coupling and edges in edge controllability.

Our findings raise many open questions. For example, is it possible to achieve partial control of a subset of edge states in GED from a minimal number of driver nodes? Could a method be developed to implement target control of GED? How to optimize edge controllability with small perturbations of coupling and network structure? What is the energy cost of controlling GED?

## Supplementary Information

Supplementary Information.

## Data Availability

The data generated and analysed during the current study are available from the corresponding author on reasonable request.
