# Supplementary material for: Structural controllability of general edge dynamics in complex network
Source: Sci Rep. 2023 Feb 28;13:3393. doi: 10.1038/s41598-023-30554-7 (PMC9974982; doi:10.1038/s41598-023-30554-7)
Supplement: Supplementary file 1 — Supplementary Information. [file 41598_2023_30554_MOESM1_ESM.pdf]

Supplementary Note 1: Structural controllability of general edge dynamics

We first introduce the minimum input theorem[1] applied to the nodal dynamics of $L(G')$. Specifically, the bipartite graph $H(G)$ of $L(G')$ is defined as $H(G) = (V_W^+ \cup V_W^-, \Gamma)$, where $V_W^+ = \{x_1^+, ..., x_M^+\}$ and $V_W^- = \{x_1^-, ..., x_M^-\}$ are the left node set and right node set corresponding to the M rows and columns of the state matrix W, respectively, and the edge set $\Gamma = \{(x_j^+, x_i^-)|w_{ij} \neq 0\}$. Then the concept of matching in $H(G)$ is as follows.

Definition 1 *For an undirected bipartite graph $H(G)$, its edge subset $\mathscr{M}$ is a matching if all edges in $\mathscr{M}$ do not have common nodes with each other. A node in the right node set is matched if it is incident to an edge in $\mathscr{M}$. Otherwise, it is an unmatched node.*

A matching of maximum size is called a maximum matching. A maximum matching is called perfect if all nodes are matched. The maximum matching of a bipartite graph can be determined by the Hopcroft-Karp algorithm[2], which runs in $O(\sqrt{V}E)$ time. Note that in general there could be many different maximum matchings with the same size for a given graph. Based on the maximum matching, we introduce the lemma as follows.

Lemma 1 *The minimum number of driver nodes need to fully control the nodal dynamics of $L(G')$ is one if there is a perfect matching in its bipartite graph $H(A)$. Otherwise, it equals the number of unmatched nodes with respective to any maximum matchings. In this case, the driver nodes are just the unmatched nodes.*

Now we propose a framework to determine the minimum driver nodes and driven edges required to control GED. We divide $H(G)$ into N matching blocks based on N switching matrices. The matching block corresponding to a switching matrix S_v contains the incoming edge states and outgoing edges edge states of the node v, where the incoming edge states and outgoing edges edge correspond to the left and right nodes of the matching block, respectively. Meanwhile the independent free parameters in S_v correspond one-to-one to the edges in the matching block.

Remark 1 *Each matching block is independent, i.e., there is no edge linking the nodes from two different matching blocks. The left and right node sets of N matching blocks just cover all the incoming and outgoing edge states of G.*

Then we give the lemma as follows.

Lemma 2 *The matching block of S_v contains unmatched nodes if and only if S_v has no full row-rank, i.e.,* $\text{rank}_\text{g}(S_v) < k_v^+$. *The number of unmatched nodes in the matching block of S_v is equal to* $k_v^+ - \text{rank}_\text{g}(S_v)$.

Proof 1 *According to Remark 1, we can prove the lemma by one matching block and its corresponding switching matrix. We first prove the sufficiency. For a node v with $k_v^+ \times k_v^-$ switching matrix S_v, the left and right node sets of its matching block correspond to the number of columns and rows of S_v, respectively. Any two matching edges in a matching block correspond to two independent free parameters with different rows and columns in S_v. These two parameters are irreducible and will add* 1 *to the generic rank of S_v, respectively. So the maximum number of matching edges of a matching block is equal to the generic rank of S_v. When* $\text{rank}_\text{g}(S_v) < k_v^+$, *the number of matching edges is less than the number of nodes in the right node set, resulting in unmatched nodes in the matching block. Meanwhile, the number of unmatched nodes in the matching block of S_v is equal to* $k_v^+ - \text{rank}_\text{g}(S_v)$.

Then we prove the necessity. When the matching block contains unmatched nodes, the number of matching edges is less than the number of nodes in the right node set. Since the maximum number of matching edges of a matching block is equal to the generic rank of S_v, the matching block contains unmatched nodes that can deduce $\text{rank}_\text{g}(S_v) < k_v^+$.

Combining Lemma 1 and 2, we can prove the main conclusions described by Eqs. (4) and (5). The proof process is as follows.

Proof 2 *Eq. (2) indicates that the GED of a digraph G is equivalent to the nodal dynamics of its trimmed line graph $L(G')$. They have the same state set and state matrix W. The equivalence shows that the driver nodes in $L(G')$ correspond one-to-one with the driven edges in G. Applying the minimum input theorem to the nodal dynamics of $L(G')$ gives us the bipartite graph $H(G)$. Then the maximum matching method can determine the unmatched nodes in $H(G)$, which correspond to the driver nodes required to control the nodal dynamics of $L(G')$, and correspond to the driven edges required to control the GED of G. The maximum matching method determines the minimum number of unmatched nodes in $H(G)$. Accordingly, the number of driven edges required to control the GED of G is also minimal. Meanwhile, Eq. (2) indicates that the driver node for controlling GED*

of G is the starting node of the driven edge. Therefore, we can determine the minimum number of driver nodes and driven edges required to control the GED of G.

We divide $H(G)$ into N matching blocks based on N switching matrices. According to Remark 1, we can give the proof with one matching block and its corresponding switching matrix. According to Lemma 2, a matching block of S_v contains unmatched nodes if and only if S_v has no full row-rank. Therefore, a node v is the driver node if and only if $\mathrm{rank_g}(S_v) < k_v^+$. Accumulate N nodes to get $N_{(\mathrm{rank_g}(S_v)<k_v^+)}$. To ensure reachability, we randomly select a node in each full-rank component to apply the external input. Since the input matrix H of the GED is a diagonal matrix, the nodes to which the external input is applied are all driver nodes. In summary, the minimum number of drive nodes required to control the GED is $N_\mathrm{D} = N_{(\mathrm{rank_g}(S_v)<k_v^+)} + \sum_{i=1}^{N_\beta} \beta_i$.

According to Lemma 2, if a node v is the driver node, the number of unmatched nodes in the matching block of S_v is equal to $k_v^+ - \mathrm{rank_g}(S_v)$. Therefore, the outgoing edge set of the driver node v contains $k_v^+ - \mathrm{rank_g}(S_v)$ driven edges. Accumulate N nodes to get $\sum_{i=1}^{N}(k_i^+ - \mathrm{rank_g}(S_i))$. To ensure reachability, we randomly select an outgoing edge of the driver node in each full-rank component as the driven edge. In summary, the minimum number of driven edges required to control the GED is $M_\mathrm{D} = \sum_{i=1}^{N}(k_i^+ - \mathrm{rank_g}(S_i)) + \sum_{i=1}^{N_\beta} \beta_i$.

Supplementary Note 2: Analytical results

The dependence of edge controllability on local network information allows us to derive analytical results in terms of the coupling density P and the joint degree distribution $P(k_v^- = i, k_v^+ = j) = P_{ij}$. Firstly, we calculate, by statistical methods, the probability that a $k_v^+ \times k_v^-$ switching matrix satisfies $\mathrm{rank_g}(S_v) = k_v^+$ under the given P. Specifically, $P \in [0,1]$ quantifies the probability that an element in the switching matrix is an independent free parameter. For a given $k_v^+ \times k_v^-$ all-zero switching matrix S_v, we determine whether each element is 0 or an independent free parameter based on the given P. Then we calculate the generic rank $\mathrm{rank_g}(S_v)$ of the switching matrix and judge whether it satisfies $\mathrm{rank_g}(S_v) = k_v^+$. Through 1000 such independent simulations, the probability $P_{i,j}^\mathrm{N}$ that the switching matrix $(k_v^- = i, k_v^+ = j)$ satisfies $\mathrm{rank_g}(S_v) = k_v^+$ under the given P is calculated. Secondly, we compute $k_v^+ - \mathrm{rank_g}(S_v)$ in each independent simulation. The mean $P_{i,j}^\mathrm{M}$ of 1000 independent simulations is an estimate of the number of driven edges for the $k_v^+ \times k_v^-$ switching matrix under the given P. Thirdly, we compute the proportion $\mathrm{rank_g}(S_v)/(k_v^+ k_v^- P)$ of effective coupling in each independent simulation. The mean $P_{i,j}^\mathrm{E}$ of 1000 independent simulations is an estimated proportion of effective coupling for the $k_v^+ \times k_v^-$ switching matrix under the given P. Finally, we calculate the proportion of three kinds of edges based on their judgment method in each independent simulation, and get the estimated proportions of critical $P_{i,j}^\mathrm{C}$, ordinary $P_{i,j}^\mathrm{O}$ and intermittent $P_{i,j}^\mathrm{I}$ edges for the $k_v^+ \times k_v^-$ switching matrix under the given P.

We neglect the possible presence of full-rank component, which is uncommon in directed networks and has little effect on N_D and M_D. Meanwhile, we assume that in- and out-degrees of each node are uncorrelated to offer analytical results. The divergent $(k_v^+ > k_v^-)$ node must not satisfy $\mathrm{rank_g}(S_v) = k_v^+$, and the balanced $(k_v^+ = k_v^-)$ and convergent $(k_v^+ < k_v^-)$ nodes may satisfy $\mathrm{rank_g}(S_v) = k_v^+$. Therefore, the proportion n_D of driver nodes is given as

$$n_\mathrm{D} = 1 - \sum_{i=0}^{\infty} P_{i,0} - \sum_{i=1}^{\infty}\sum_{j=0}^{\infty} P_{(i+j),i} P_{(i+j),i}^\mathrm{N}. \tag{7}$$

Then the proportion m_D of driven edges is

$$m_\mathrm{D} = \frac{1}{\langle k \rangle}\sum_{i=1}^{\infty}\sum_{j=1}^{\infty} P_{i,j} P_{i,j}^\mathrm{M} + \frac{1}{\langle k \rangle}\sum_{i=1}^{\infty} i P_{0,i}, \tag{8}$$

where the average degree is $\langle k \rangle = \langle k^- \rangle = \langle k^+ \rangle = M/N$. When $P = 1$, all the elements in the switching matrix of each node are independent free parameters, n_D and m_D reach the lower bounds. Their analysis results have been given in[3]. Specifically, the lower bound of driver nodes is $n_\mathrm{D}^\mathrm{L} = \frac{1}{2}(1 - \sum_{i=0}^{\infty} P_{ii})$ and the lower bound of driven edges is $m_\mathrm{D}^\mathrm{L} = \frac{1}{\langle k \rangle}\sum_{i=0}^{\infty}\sum_{j=1}^{\infty} j P_{i,(i+j)}$. When $P = 0$, all the elements in the switching matrix of each node are 0, the n_D and m_D reach the upper bound. The upper bound of driver nodes is $n_\mathrm{D}^\mathrm{U} = 1 - \sum_{i=0}^{\infty} P_{i,0}$. The upper bound of driven edge is $m_\mathrm{D}^\mathrm{U} = 1$.

When P is given, the estimated proportion of effective coupling for the $k_v^+ \times k_v^-$ switching matrix is $P_{i,j}^\mathrm{E}$. Therefore, the proportion P_E of effective coupling is

$$P_\mathrm{E} = \sum_{i=1}^{\infty}\sum_{j=1}^{\infty} P_{i,j} P_{i,j}^\mathrm{E}. \tag{9}$$

When $P = 1$, the proportion of effective coupling is $P_\mathrm{E} = \sum_{i=1}^{\infty}\sum_{j=0}^{\infty} \frac{P_{i+j,i}}{i+j} + \sum_{i=1}^{\infty}\sum_{j=1}^{\infty} \frac{P_{i,i+j}}{i+j}$. When $P = 0$, we have $P_\mathrm{E} = 0$.

The outgoing edges of the nodes with $k_v^+ > k_v^- = 0$ are all critical edges. Meanwhile, when P is given, the estimated proportion of critical edges for the $k_v^+ \times k_v^-$ switching matrix is $P^{\mathrm{C}}_{i,j}$. Therefore, the proportion m_{C} of critical edges is

$$m_{\mathrm{C}} = \frac{1}{\langle k\rangle}\sum_{i=1}^{\infty} iP_{0,i} + \frac{1}{\langle k\rangle}\sum_{i=1}^{\infty}\sum_{j=1}^{\infty} P_{i,j}P^{\mathrm{C}}_{i,j}. \tag{10}$$

Similarly, the proportion m_{O} of ordinary edges is

$$m_{\mathrm{O}} = \frac{1}{\langle k\rangle}\sum_{i=1}^{\infty}\sum_{j=1}^{\infty} P_{i,j}P^{\mathrm{O}}_{i,j}. \tag{11}$$

Then the proportion m_{I} of intermittent edges is

$$m_{\mathrm{I}} = \frac{1}{\langle k\rangle}\sum_{i=1}^{\infty}\sum_{j=1}^{\infty} P_{i,j}P^{\mathrm{I}}_{i,j}. \tag{12}$$

When $P = 1$, the proportion of critical edge is $m_{\mathrm{C}} = \frac{1}{\langle k\rangle}\sum_{i=1}^{\infty} P_{0,i}i$, the proportion of ordinary edge is $m_{\mathrm{O}} = \frac{1}{\langle k\rangle}\sum_{i=1}^{\infty}\sum_{j=0}^{\infty} P_{(i+j),i}i$, and the proportion of intermittent edge is $m_{\mathrm{I}} = \frac{1}{\langle k\rangle}\sum_{i=1}^{\infty}\sum_{j=1}^{\infty} P_{i,(i+j)}(i+j)$. When $P = 0$, all the elements in the switching matrix of each node are 0, causing $m_{\mathrm{C}} = 1$, $m_{\mathrm{O}} = 0$ and $m_{\mathrm{I}} = 0$.

The ER networks are generated by static model[4], where both the in- and out-degrees follow a Poisson distribution.

$$P(k_v^+ = k) = P(k_v^- = k) = \frac{(\langle k\rangle^k e^{-\langle k\rangle})}{k!}. \tag{13}$$

By substituting the Poisson distribution into Eq. (7), we obtain

$$n_{\mathrm{D}} = 1 - \sum_{i=0}^{\infty}\frac{\langle k\rangle^i e^{-2\langle k\rangle}}{i!} - \sum_{i=1}^{\infty}\sum_{j=0}^{\infty}\frac{\langle k\rangle^{2i+j}e^{-2\langle k\rangle}}{i!(i+j)!}P^{\mathrm{N}}_{(i+j),i} = 1 - e^{-\langle k\rangle} - e^{-2\langle k\rangle}\sum_{i=1}^{\infty}\sum_{j=0}^{\infty}\frac{\langle k\rangle^{2i+j}}{i!(i+j)!}P^{\mathrm{N}}_{(i+j),i}. \tag{14}$$

Similarly, the analysis result of m_{D} is

$$m_{\mathrm{D}} = \frac{e^{-2\langle k\rangle}}{\langle k\rangle}\sum_{i=1}^{\infty}\sum_{j=1}^{\infty}\frac{\langle k\rangle^{i+j}}{i!j!}P^{\mathrm{M}}_{i,j} + \frac{e^{-2\langle k\rangle}}{\langle k\rangle}\sum_{i=1}^{\infty} i\frac{\langle k\rangle^i}{i!} = \frac{e^{-2\langle k\rangle}}{\langle k\rangle}\sum_{i=1}^{\infty}\sum_{j=1}^{\infty}\frac{\langle k\rangle^{i+j}}{i!j!}P^{\mathrm{M}}_{i,j} + e^{-\langle k\rangle}. \tag{15}$$

When $P = 1$, the lower bounds[3] are $n^{\mathrm{L}}_{\mathrm{D}} = \frac{1}{2}(1 - e^{-2\langle k\rangle}I_0(2\langle k\rangle))$ and $m^{\mathrm{L}}_{\mathrm{D}} = \frac{e^{-2\langle k\rangle}}{\langle k\rangle}\sum_{j=1}^{\infty} jI_j(2\langle k\rangle)$, respectively, where $I_a(x)$ is the modified Bessel function of the first kind. When $P = 0$, the upper bounds are $n^{\mathrm{U}}_{\mathrm{D}} = 1 - e^{-\langle k\rangle}$ and $m^{\mathrm{U}}_{\mathrm{D}} = 1$, respectively. The analysis result of P_{E} is

$$P_{\mathrm{E}} = e^{-2\langle k\rangle}\sum_{i=1}^{\infty}\sum_{j=1}^{\infty}\frac{\langle k\rangle^{i+j}}{i!j!}P^{\mathrm{E}}_{i,j}. \tag{16}$$

When $P = 1$, we have $P_{\mathrm{E}} = e^{-2\langle k\rangle}\sum_{i=1}^{\infty}\frac{\langle k\rangle^{2i}HF[(1,i),(1+i,1+i),\langle k\rangle]}{i(i!)^2} + e^{-2\langle k\rangle}\sum_{i=1}^{\infty}\frac{\langle k\rangle^{1+2i}HF[(1,1+i),(2+i,2+i),\langle k\rangle]}{(1+i)i!(1+i)!}$, where $HF[(a_1,...,a_p),(b_1,...,b_q),z]$ is the generalized hypergeometric function. When $P = 0$, we have $P_{\mathrm{E}} = 0$. The analysis result of critical edge is

$$m_{\mathrm{C}} = \frac{1}{\langle k\rangle}\sum_{i=1}^{\infty} i\frac{\langle k\rangle^i}{i!}e^{-2\langle k\rangle} + \frac{e^{-2\langle k\rangle}}{\langle k\rangle}\sum_{i=1}^{\infty}\sum_{j=1}^{\infty}\frac{\langle k\rangle^{i+j}}{i!j!}P^{\mathrm{C}}_{i,j} = e^{-\langle k\rangle} + \frac{e^{-2\langle k\rangle}}{\langle k\rangle}\sum_{i=1}^{\infty}\sum_{j=1}^{\infty}\frac{\langle k\rangle^{i+j}}{i!j!}P^{\mathrm{C}}_{i,j}. \tag{17}$$

Similarly, the analysis result of ordinary edge is

$$m_{\mathrm{O}} = \frac{e^{-2\langle k\rangle}}{\langle k\rangle}\sum_{i=1}^{\infty}\sum_{j=1}^{\infty}\frac{\langle k\rangle^{i+j}}{i!j!}P^{\mathrm{O}}_{i,j}. \tag{18}$$

Then the analysis result of intermittent edge is

$$m_{\mathrm{I}} = \frac{e^{-2\langle k\rangle}}{\langle k\rangle}\sum_{i=1}^{\infty}\sum_{j=1}^{\infty}\frac{\langle k\rangle^{i+j}}{i!j!}P^{\mathrm{I}}_{i,j}. \tag{19}$$

When $P=1$, we have $m_{\mathrm{C}}=e^{-<k>}$, $m_{\mathrm{O}}=e^{-2\langle k\rangle}\sum_{j=1}^{\infty}I_j(2\langle k\rangle)$ and $m_{\mathrm{I}}=\frac{e^{-2\langle k\rangle}}{\langle k\rangle}\sum_{j=1}^{\infty}(i+j)I_j(2\langle k\rangle)-e^{-\langle k\rangle}$. When $P=0$, we have $m_{\mathrm{C}}=1$, $m_{\mathrm{O}}=0$ and $m_{\mathrm{I}}=0$.

The EX networks with an exponential degree distribution are generated by configuration model[5]. Both the in- and out-degrees follow the same exponential distribution, which is

$$P(k_v^+=k)=P(k_v^-=k)=Ce^{-k/\kappa}, \tag{20}$$

where $C=1-e^{-1/\kappa}$ and $\kappa=1/\log\frac{1+\langle k\rangle}{\langle k\rangle}$. By substituting the exponential distribution into Eq. (7), we obtain

$$n_{\mathrm{D}}=1-C^2\sum_{i=0}^{\infty}e^{-i/\kappa}-C^2\sum_{i=1}^{\infty}\sum_{j=0}^{\infty}e^{-(2i+j)/\kappa}P^{\mathrm{N}}_{(i+j),i}=\frac{\langle k\rangle}{\langle k\rangle+1}-C^2\sum_{i=1}^{\infty}\sum_{j=0}^{\infty}e^{-(2i+j)/\kappa}P^{\mathrm{N}}_{(i+j),i}. \tag{21}$$

Similarly, the analysis result of m_{D} is

$$\begin{aligned} m_{\mathrm{D}}&=\frac{C^2}{\langle k\rangle}\sum_{i=1}^{\infty}\sum_{j=1}^{\infty}e^{-(i+j)/\kappa}P^{\mathrm{M}}_{i,j}+\frac{C^2}{\langle k\rangle}\sum_{i=1}^{\infty}ie^{-i/\kappa}\\ &=\frac{C^2}{\langle k\rangle}\sum_{i=1}^{\infty}\sum_{j=1}^{\infty}e^{-(i+j)/\kappa}P^{\mathrm{M}}_{i,j}+\frac{1}{\langle k\rangle+1}.\end{aligned} \tag{22}$$

When $P=1$, the lower bounds[3] are $n^{\mathrm{L}}_{\mathrm{D}}=\frac{\langle k\rangle}{2\langle k\rangle+1}$ and $m^{\mathrm{L}}_{\mathrm{D}}=\frac{\langle k\rangle+1}{2\langle k\rangle+1}$, respectively. When $P=0$, the upper bounds are $n^{\mathrm{U}}_{\mathrm{D}}=\frac{\langle k\rangle}{\langle k\rangle+1}$ and $m^{\mathrm{U}}_{\mathrm{D}}=1$, respectively. The analysis result of P_{E} is

$$P_{\mathrm{E}}=C^2\sum_{i=1}^{\infty}\sum_{j=1}^{\infty}e^{-(i+j)/\kappa}P^{\mathrm{E}}_{i,j}. \tag{23}$$

When $P=1$, we have $P_{\mathrm{E}}=\frac{-2\langle k\rangle log(\frac{1}{1+\langle k\rangle})}{(1+\langle k\rangle)^2}+\frac{(1+2\langle k\rangle)log(\frac{1+2\langle k\rangle}{(1+\langle k\rangle)^2})}{(1+\langle k\rangle)^2}$. When $P=0$, we have $P_{\mathrm{E}}=0$. The analysis result of the critical edge is

$$m_{\mathrm{C}}=\frac{1}{\langle k\rangle}C^2\sum_{i=1}^{\infty}ie^{-i/\kappa}+\frac{C^2}{<k>}\sum_{i=1}^{\infty}\sum_{j=1}^{\infty}e^{-(i+j)/\kappa}P^{\mathrm{C}}_{i,j}=\frac{1}{\langle k\rangle+1}+\frac{C^2}{\langle k\rangle}\sum_{i=1}^{\infty}\sum_{j=1}^{\infty}e^{-(i+j)/\kappa}P^{\mathrm{C}}_{i,j}. \tag{24}$$

The analysis result of the ordinary edge is

$$m_{\mathrm{O}}=\frac{C^2}{\langle k\rangle}\sum_{i=1}^{\infty}\sum_{j=1}^{\infty}e^{-(i+j)/\kappa}P^{\mathrm{O}}_{i,j}. \tag{25}$$

The analysis result of the intermittent edge is

$$m_{\mathrm{I}}=\frac{C^2}{\langle k\rangle}\sum_{i=1}^{\infty}\sum_{j=1}^{\infty}e^{-(i+j)/\kappa}P^{\mathrm{I}}_{i,j}. \tag{26}$$

When $P=1$, we have $m_{\mathrm{C}}=\frac{1}{\langle k\rangle+1}$, $m_{\mathrm{O}}=\frac{\langle k\rangle(\langle k\rangle+1)}{(2\langle k\rangle+1)^2}$ and $m_{\mathrm{I}}=\frac{\langle k\rangle^2(3\langle k\rangle+2)}{(\langle k\rangle+1)(2\langle k\rangle+1)^2}$. When $P=0$, we have $m_{\mathrm{C}}=1$, $m_{\mathrm{O}}=0$ and $m_{\mathrm{I}}=0$.

The SF networks are generated by static model[4]. Both the in- and out-degrees follow a power-law distribution[6], i.e.,

$$P(k_v^+=k)=P(k_v^-=k)=\frac{[\langle k\rangle(1-a)]^{\frac{1}{a}}}{a}\frac{\Gamma(k-1/a,\langle k\rangle(1-a))}{\Gamma(k+1)}, \tag{27}$$

where $\Gamma(x,y)$ is the incomplete Gamma function. Let δ denote $\frac{[\langle k\rangle(1-a)]^{\frac{1}{a}}}{a}$ and let Γ_k denote $\frac{\Gamma(k-1/a,\langle k\rangle(1-a))}{\Gamma(k+1)}$. By substituting the power-law distribution into Eq. (7), we obtain

$$n_{\mathrm{D}}=1-\delta^2\sum_{i=0}^{\infty}\Gamma_i\Gamma_0-\delta^2\sum_{i=1}^{\infty}\sum_{j=0}^{\infty}\Gamma_i\Gamma_{i+j}P^{\mathrm{N}}_{(i+j),i}=1-\delta\Gamma_0-\delta^2\sum_{i=1}^{\infty}\sum_{j=0}^{\infty}\Gamma_i\Gamma_{i+j}P^{\mathrm{N}}_{(i+j),i}. \tag{28}$$

Similarly, the analysis result of m_{D} is

$$m_{\mathrm{D}} = 1 - \frac{\delta^2}{\langle k \rangle} \sum_{i=1}^{\infty} \sum_{j=1}^{\infty} \Gamma_i \Gamma_j (j - P_{i,j}^{\mathrm{M}}). \tag{29}$$

When $P = 1$, the lower bounds[3] are $n_{\mathrm{D}}^{\mathrm{L}} = \frac{1}{2}(1 - \delta^2 \sum_{i=0}^{\infty} \Gamma_i^2)$ and $m_{\mathrm{D}}^{\mathrm{L}} = \frac{\delta^2}{\langle k \rangle} \sum_{i=0}^{\infty} \sum_{j=1}^{\infty} j \Gamma_i \Gamma_{i+j}$, respectively. When $P = 0$, the upper bounds are $n_{\mathrm{D}}^{\mathrm{U}} = 1 - \delta \Gamma_0$ and $m_{\mathrm{D}}^{\mathrm{U}} = 1$, respectively. The analysis result of P_{E} is

$$P_{\mathrm{E}} = \delta^2 \sum_{i=1}^{\infty} \sum_{j=1}^{\infty} \Gamma_i \Gamma_j P_{i,j}^{\mathrm{E}}. \tag{30}$$

When $P = 1$, we have $P_{\mathrm{E}} = \delta^2 \sum_{i=1}^{\infty} \sum_{j=0}^{\infty} \frac{\Gamma_{i+j} \Gamma_i}{i+j} + \delta^2 \sum_{i=1}^{\infty} \sum_{j=1}^{\infty} \frac{\Gamma_i \Gamma_{i+j}}{i+j}$. When $P = 0$, we have $P_{\mathrm{E}} = 0$. The analysis result of the critical edge is

$$m_{\mathrm{C}} = \frac{\delta^2}{\langle k \rangle} \sum_{i=1}^{\infty} i \Gamma_0 \Gamma_i + \frac{\delta^2}{\langle k \rangle} \sum_{i=1}^{\infty} \sum_{j=1}^{\infty} \Gamma_i \Gamma_j P_{i,j}^{\mathrm{C}}. \tag{31}$$

The analysis result of the ordinary edge is

$$m_{\mathrm{O}} = \frac{\delta^2}{\langle k \rangle} \sum_{i=1}^{\infty} \sum_{j=1}^{\infty} \Gamma_i \Gamma_j P_{i,j}^{\mathrm{O}}. \tag{32}$$

The analysis result of the intermittent edge is

$$m_{\mathrm{I}} = \frac{\delta^2}{\langle k \rangle} \sum_{i=1}^{\infty} \sum_{j=1}^{\infty} \Gamma_i \Gamma_j P_{i,j}^{\mathrm{I}}. \tag{33}$$

When $P = 1$, we have $m_{\mathrm{C}} = \frac{\delta^2}{\langle k \rangle} \sum_{i=1}^{\infty} i \Gamma_0 \Gamma_i$, $m_{\mathrm{O}} = \frac{\delta^2}{\langle k \rangle} \sum_{i=1}^{\infty} \sum_{j=0}^{\infty} i \Gamma_i \Gamma_{i+j}$ and $m_{\mathrm{I}} = \frac{\delta^2}{\langle k \rangle} \sum_{i=1}^{\infty} \sum_{j=1}^{\infty} (i+j) \Gamma_i \Gamma_{i+j}$. When $P = 0$, we have $m_{\mathrm{C}} = 1$, $m_{\mathrm{O}} = 0$ and $m_{\mathrm{I}} = 0$.

Supplementary Note 3: Real network analysis results

We substantiate how to derive theoretical predictions of real networks. Specifically, we insert the coupling density, probability $P_{i,j}^{\mathrm{N}}$ and degree distribution of a real network into Eq. (7) to predict the fraction of driver nodes $n_{\mathrm{D}}^{\mathrm{analytic}}$ via

$$n_{\mathrm{D}}^{\mathrm{analytic}} = 1 - \sum_{i=0}^{\infty} P_{\mathrm{in}}(i) P_{\mathrm{out}}(0) - \sum_{i=1}^{\infty} \sum_{j=0}^{\infty} P_{\mathrm{in}}(i+j) P_{\mathrm{out}}(i) P_{(i+j),i}^{\mathrm{N}}, \tag{34}$$

where $P_{\mathrm{in}}(x)$ and $P_{\mathrm{out}}(y)$ are the fraction of nodes in the real network with degree $k_v^- = x$ and $k_v^+ = y$, respectively. The theoretical predictions of the fraction of driven edges $m_{\mathrm{D}}^{\mathrm{analytic}}$ and three kinds of edges: $m_{\mathrm{C}}^{\mathrm{analytic}}$, $m_{\mathrm{O}}^{\mathrm{analytic}}$, $m_{\mathrm{I}}^{\mathrm{analytic}}$ are given in a similar way.

References

1. Liu, Y. Y., Slotine, J. J. & Barabási, A. L. Controllability of complex networks. *nature* **473**, 167–173, DOI: https://doi.org/10.1038/nature10011 (2011).

2. Hopcroft, J. E. & Karp, R. M. An nˆ5/2 algorithm for maximum matchings in bipartite graphs. *SIAM J. on computing* **2**, 225–231, DOI: https://doi.org/10.1137/0202019 (1973).

3. Nepusz, T. & Vicsek, T. Controlling edge dynamics in complex networks. *Nat. Phys.* **8**, 568–573, DOI: https://doi.org/10.1038/Nphys2327 (2012).

4. Goh, K. I., Kahng, B. & Kim, D. Universal behavior of load distribution in scale-free networks. *Phys. review letters* **87**, 278701, DOI: https://doi.org/10.1103/PhysRevLett.87.278701 (2001).

5. Chung, F. & Lu, L. Connected components in random graphs with given expected degree sequences. *Annals combinatorics* **6**, 125–145, DOI: https://doi.org/10.1007/PL00012580 (2002).

6. Catanzaro, M. & Pastor Satorras, R. Analytic solution of a static scale-free network model. *The Eur. Phys. J. B-Condensed Matter Complex Syst.* **44**, 241–248, DOI: https://doi.org/10.1140/epjb/e2005-00120-9 (2005).
